# ﻿First karyotype description of the species of *Adenomera* Steindachner, 1867 (Anura, Leptodactylidae) in the “ *thomei*” clade

**DOI:** 10.3897/compcytogen.v16.i3.82641

**Published:** 2022-08-30

**Authors:** Ramon Costa Dominato, Guilherme Costa de Oliveira, Carla Santana Cassini, Victor Goyannes Dill Orrico, Cléa dos Santos Ferreira Mariano, Janisete Gomes Silva

**Affiliations:** 1 Programa de Pós-Graduação em Genética e Biologia Molecular, Departamento de Ciências Biológicas, Universidade Estadual de Santa Cruz, Rodovia Jorge Amado, Km 16. CEP: 45662-900 Ilhéus, BA Brazil Universidade Estadual de Santa Cruz Ilhéus Brazil; 2 Tropical Herpetology Laboratory, Departamento de Ciências Biológicas, Universidade Estadual de Santa Cruz, Rodovia Jorge Amado, Km 16. CEP: 45662-900 Ilhéus, BA Brazil Universidade Estadual de Santa Cruz Ilhéus Brazil; 3 Programa de Pós-Graduação em Zoologia, Universidade Estadual de Santa Cruz, Departamento de Ciências Biológicas, Rodovia Jorge Amado, Km 16. CEP 45662-900 Ilhéus, BA Brazil Universidade Estadual de Santa Cruz Ilhéus Brazil; 4 Departamento de Ciências Biológicas, Universidade Estadual de Santa Cruz, Rodovia Jorge Amado, Km 16. CEP 45662-900 Ilhéus, BA Brazil Universidade Estadual de Santa Cruz Ilhéus Brazil; 5 Laboratório de Artrópodes Sociais - LABAS Departamento de Ciências Biológicas, Universidade Estadual de Santa Cruz, Rodovia Jorge Amado, Km 16. CEP: 45662-900 Ilhéus, BA Brazil Universidade Estadual de Santa Cruz Ilhéus Brazil

**Keywords:** Chromosomes, cryptic species, cytogenetics, Giemsa, taxonomy

## Abstract

The genus *Adenomera* Steindachner, 1867 currently comprises 29 nominal species, some of which are suggested to be cryptic species complexes. The present study was carried out with specimens of the “*thomei*” clade that encompasses three taxa distributed in the Atlantic Forest biome: *Adenomerathomei* Almeida et Angulo, 2006, *Adenomera* sp. L., and *Adenomera* sp. M. We used classical cytogenetics to describe the diploid number and karyomorphology of these three species collected in two different locations in the state of Bahia, Brazil. Our results revealed the diploid number 2n = 24 (FN = 34) with two pairs of metacentric chromosomes (pairs 1 and 5), three pairs of submetacentric chromosomes (pairs 2, 3, and 4), and seven pairs of telocentric chromosomes (pairs 6, 7, 8, 9, 10, 11, and 12). Further morphological, bioacoustic, and cytogenetic data (C-banding and AgNor) are needed to better delineate the lineages within the “*thomei*” clade.

## ﻿Introduction

The genus *Adenomera* Steindachner, 1867 currently comprises 29 described species that are distributed from tropical South America to the east of the Andean region (Carvalho et al. 2021). Due to the history of systematic reviews and the complex taxonomy of this group, taxonomic knowledge has not kept pace with the knowledge on its phylogeny (Duellman 2005; Menin et al. 2008; Fouquet et al. 2014). Out of the several hurdles for taxonomic studies on this genus, we highlight the high intra and interspecific similarities and the presence of cryptic species complexes (Fouquet et al. 2014). The difficulty increases when studies use only molecular data, disregarding other characteristics and making the interpretation of results less accurate (Pyron and Wiens 2011; De Sá et al. 2014).

Cytogenetic studies on the genus *Adenomera* date from the 1970s (Bogart 1970, 1974) when the karyotypes of *Adenomeraandreae* (Müller, 1923), *Adenomerahylaedactyla* (Cope, 1868), *Adenomeralutzi* Heyer, 1975, and *Adenomeramarmorata* (Steindachner, 1867) were described. However, the volume of cytogenetic information for the genus has not significantly advanced over these five decades. Campos et al. (2009) described the karyotypes of individuals from western São Paulo associating them with the nominal species Adenomeraaff.bokermanni Heyer, 1973, *A.hylaedactyla*, and *A.marmorata*. Additionally, the karyotype of *Adenomeradiptyx* (Boettger, 1885) was described by Zaracho and Hernando (2011). Thus, there is cytogenetic information for only five species among the 29 species described for this genus. Therefore, the small number of described karyotypes makes it difficult to both understand the chromosomal evolution of the genus and to better delimit species (Campos et al. 2009; Zaracho and Hernando 2011).

Among the clades within the genus *Adenomera*, the species of the *thomei* clade, *Adenomerathomei*Almeida and Angulo (2006), *Adenomera* sp. L, and *Adenomera* sp. M, are restricted to the Atlantic Forest in Brazil. *Adenomerathomei* was described from specimens collected in a cocoa plantation in the municipality of Linhares in the state of Espírito Santo (Almeida and Angulo 2006). Currently, there are records of this species also in the states of Rio de Janeiro, São Paulo, Minas Gerais, and Bahia (Almeida and Angulo 2006; Fouquet et al. 2014). The specific boundaries among these lineages are unclear mainly due to the lack of information on *Adenomera* sp. L and *Adenomera* sp. M, both of which are found only in the southern region of the state of Bahia (Fouquet et al. 2014). Knowledge on the bioacoustics, morphology, and cytogenetics for representatives of this clade is scarce (Angulo et al. 2003; Angulo 2004; Duellman 2005) and thus far it has not been used to distinguish between these two lineages.

Karyotypic information associated to DNA sequence data has helped clarify the taxonomy and systematics of some Brazilian anuran groups (Lourenço et al. 2008; Targueta et al. 2010; Suárez et al. 2013; Lourenço et al. 2015; Ferro et al. 2016; Marciano-Jr et al. 2021). To date, all information available regarding cytogenetic data within *Adenomera* populations is taxonomically inconclusive (e.g., Campos et al. 2009). Nevertheless, these chromosome data provided support on taxonomic decisions on a broad study of species delimitation of *Adenomeramarmorata*, which included DNA sequence, morphological, and bioacoustic data (Cassini et al. 2020). Thus, it is clear that further cytogenetic studies on the genus *Adenomera* will allow more robust conclusions regarding this taxonomically challenging group. The objective of this study was to describe for the first time the karyotype of *Adenomera* species of the “*thomei*” clade from different locations in southern Bahia and compare the chromosomal patterns among the specimens.

## ﻿Material and methods

Cytogenetic analysis was performed using 12 specimens of two species in the “*thomei*” clade collected in three sites in the state of Bahia (BA) (Table 1) under the SISBIO license 62181. The specimens were taken to the Tropical Herpetology Laboratory at the Universidade Estadual de Santa Cruz (UESC), Ilhéus, Bahia, Brazil. We identified the specimens collected in the municipality of Ilhéus as Adenomeracf.thomei, since the bioacoustic data showed the same pattern as that recorded for populations in the “*thomei*” clade.

**Table 1.** Information on *Adenomera* specimens in the “*thomei*” clade used in this study.

Voucher	Genus	Species	Sex	Locality	Coordinates
MZUESC 22146	*Adenomera*	cf.thomei	Juvenile	Ilhéus - BA	-14.800189, -39.154594
MZUESC 22147	*Adenomera*	cf.thomei	Juvenile	Ilhéus - BA	-14.800189, -39.154594
MZUESC 22148	*Adenomera*	cf.thomei	Juvenile	Ilhéus - BA	-14.795269, -39.037339
MZUESC 22149	*Adenomera*	cf.thomei	Male	Ilhéus - BA	-14.795269, -39.037339
MZUESC 22150	*Adenomera*	cf.thomei	Male	Ilhéus - BA	-14.795269, -39.037339
MZUESC 22151	*Adenomera*	sp. L	Male	Igrapiúna - BA	-13.821933, -39.171175
MZUESC 22152	*Adenomera*	sp. L	Juvenile	Igrapiúna - BA	-13.821933, -39.171175
MZUESC 22153	*Adenomera*	sp. L	Male	Igrapiúna - BA	-13.821933, -39.171175
MZUESC 22154	*Adenomera*	sp. L	Female	Igrapiúna - BA	-13.821933, -39.171175
MZUESC 22155	*Adenomera*	sp. L	-	Igrapiúna - BA	-13.821933, -39.171175
MZUESC 22156	*Adenomera*	sp. L	-	Igrapiúna - BA	-13.821933, -39.171175
MZUESC 22157	*Adenomera*	sp. L	Juvenile	Igrapiúna - BA	-13.821933, -39.171175

We followed the protocol of Schmid (1978) with modifications. In the present study, a 2% colchicine solution (0.1 ml/10 g of weight) was used during 4–6 h. Subsequently, the specimens were sacrificed with lidocaine gel at a concentration of 5% spread over the entire body. The vouchers were fixed in 10% formaldehyde for 24 hours, kept in 70% alcohol, and deposited at the UESC Herpetological collection.

Chromosomal preparations were obtained from intestinal cells. The intestinal epithelium was kept in a hypotonic solution (0.075 M KCL) for 40 minutes and fixed in CARNOY solution (3:1 methanol: acetic acid). Then, the cell suspensions were placed on the surface of a slide and dried at room temperature in the dark. To determine chromosome composition and the fundamental number (FN), cells were stained with 3% Giemsa for 10 minutes. Chromosomes were classified according to Green and Sessions (1991) as metacentric (M), submetacentric (SM), subtelocentric (ST), and telocentric (T) (Table 2). Results obtained were compared with cytogenetic data available in the literature. The images were captured and analyzed using an Olympus BX-51 microscope, a Q-Capture Pro image capture camera, and the Image Pro Plus software. We used Adobe Photoshop CC 2019 for the analysis and arrangement of the karyotype in descending order.

**Table 2.** *Adenomera* species with described karyotype, fundamental number and bibliographic references. Species Identification followed the taxon name used in the original contribution.

Species	Karyomorphology	Diploid number	Fundamental number	References
*A.diptyx*	1M+3SM+ 9T	26	FN = 34	Zaracho and Hernando 2011
*A.andreae*	1M+4SM+2ST, 6T	26	FN = 40	Bogart 1974
*A.lutzi*	-	26	NA	Bogart 1970 apud Kuramoto, 1990
*A.hylaedactyla*	1M+ 3SM+ 9T	26	FN = 34	Campos et al. 2009
*A.hylaedactyla*	1M+1SM+2ST+9T	26	FN = 36	Bogart 1974
*A.marmorata*	2M+1SM+2ST+7T	24	FN = 34	Bogart 1974
A.cf.marmorata	3M+3SM+6T	24	FN = 34	Campos et al. 2009
A.cf.marmorata	2M+3SM+7T	24	FN = 34	Campos et al. 2009
*Adenomera* sp. L	2M+3SM+7T	24	FN = 34	Present Study
A.cf.thomei	2M+3SM+7T	24	FN = 34	Present Study
A.cf.bokermanni	2M+3SM+1ST+4T+3NP (1M + 2T)	23	FN = 34	Campos et al. 2009

## ﻿Results

**Figure 1.**
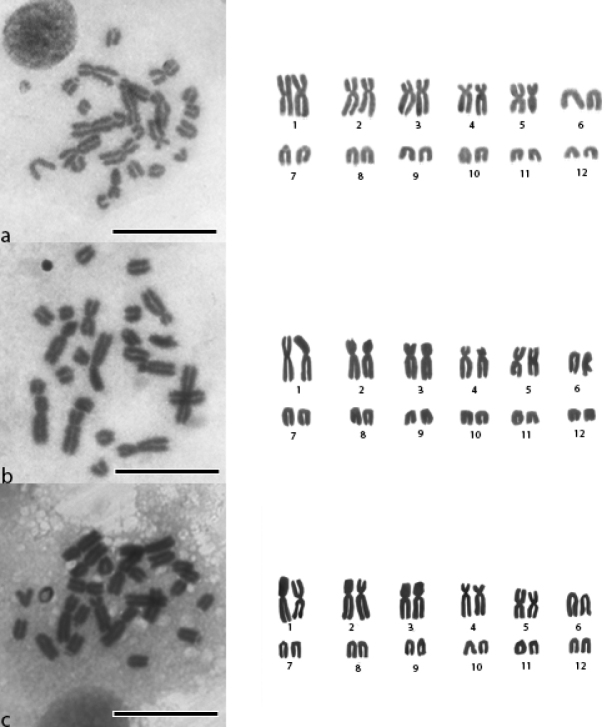
Karyotype of “*thomei*” clade specimens with conventional Giemsa staining **a**Adenomeracf.thomei from Ilhéus, Bahia, Brazil **b, c***Adenomera* sp. L from Igrapiúna, Bahia, Brazil. All specimens showed the following karyomorphology: pairs 1 and 5 metacentric, 2–4 submetacentric, and 7–2 telocentric. Scale bar: 5 μm.

We analyzed metaphases of 12 individuals of the lineages *Adenomera* sp. L (n = 6) and Adenomeracf.thomei (n = 6; sex of specimens is shown in Table 1). The karyotype of all analyzed specimens showed 2n = 24 (FN = 34) and no heteromorphic sex chromosomes. All individuals showed the karyotype 2n = 24 with a karyotypic formula of 4M + 6SM + 14T (metacentric pairs 1 and 5; submetacentric pairs 2, 3, and 4; telocentric pairs 6, 7, 8, 9, 10, 11, and 12) (Fig. 1).

The karyotypes obtained in this study and those already published for the genus *Adenomera* are shown in Table 2 with their respective diploid number, fundamental number, and karyomorphology.

## ﻿Discussion

The number of cytogenetic studies on anurans has grown in recent years (e.g., Ferro et al. 2016; Gazoni et al. 2018; Marciano-Jr et al. 2021); however, information for some families and/or genera is still scarce. The genus *Adenomera* comprises common and abundant species, some of which often occur syntopically (Cassini et al. 2020), but cytogenetic data for the entire genus are still scant compared to other anuran genera. So far, only six of the 29 described species have been karyotyped. Campos et al. (2009) analyzed four populations of *Adenomera* from the state of São Paulo and identified two species, *Adenomeramarmorata* and Adenomeraaff.bokermanni with distinct karyotypes. *Adenomeramarmorata* shows a variation in chromosome pair 12, which is metacentric in the populations of the state of São Paulo. Thus, Campos et al. (2009) hypothesized that it is an interpopulation variation, which was later confirmed by Cassini et al. (2020) in a taxonomic study on the group that integrated DNA sequences, morphology, and bioacoustics. The specimen identified by Campos et al. (2009) as A.affbokermanni was collected in the municipality of Santa Branca in the state of São Paulo, which is outside the current distribution of *A.bokermanni*, which is restricted to the southern region of the state of Paraná (Cassini et al. 2020).

The specimens analyzed in the present study were cytogenetically similar to those of *A.marmorata* and A.aff.bokermanni (Campos et al. 2009). Campos et al. (2009) found an unusual diploid number (2n = 23) when they described the karyotype of A.aff.bokermanni, Voucher - CFBH 11531, and concluded that it was most likely an indicative of a centric fusion involving the telocentric chromosome pairs 7 and 9. The authors stated that it is not possible to determine with certainty whether the differences in chromosome pairs 7 and 9 correspond to a variation restricted to the specimen analyzed. Therefore, chromosome pairs 7 and 9 will not be used for comparison in our analyses. The chromosomes of pair 8 in the specimens analyzed in the present study are telocentric, whereas those of A.aff.bokermanni are subtelocentric (Campos et al. 2009).

Furthermore, the specimens of Adenomeracf.thomei (Ilhéus, BA) and *Adenomera* sp. L (Igrapiúna, BA) in the present study showed a karyotype (2n = 24 – FN = 34) identical to that of the specimen CFBH1512 from Santa Branca (SP) and the specimen CFBH 1713 (*Adenomera* sp. J). Moreover, no bioacoustic, molecular (DNA), or morphological data are available for the Ilhéus population and a taxonomic review including all species within the clade is needed to shed light on their specific limits.

Comparative cytogenetics can be considered an important tool for recovering phylogenetic relationships and confirming taxonomic identity (e.g., Baker 1970; Silva et al. 2004; Aguiar Jr et al. 2007; Urdampilleta et al. 2013; Cassini et al. 2020). The results presented here will contribute to expand the information on the taxonomy and phylogeny of the “*thomei*” clade and consequently lead to the delimitation of its taxa.
